# Segmentation of Peripheral Nerves From Magnetic Resonance Neurography: A Fully-Automatic, Deep Learning-Based Approach

**DOI:** 10.3389/fneur.2018.00777

**Published:** 2018-09-19

**Authors:** Fabian Balsiger, Carolin Steindel, Mirjam Arn, Benedikt Wagner, Lorenz Grunder, Marwan El-Koussy, Waldo Valenzuela, Mauricio Reyes, Olivier Scheidegger

**Affiliations:** ^1^Institute for Surgical Technology and Biomechanics, University of Bern, Bern, Switzerland; ^2^Support Center for Advanced Neuroimaging, Institute for Diagnostic and Interventional Neuroradiology, Inselspital, Bern University Hospital, University of Bern, Bern, Switzerland; ^3^Department of Neurology, Inselspital, Bern University Hospital, University of Bern, Bern, Switzerland

**Keywords:** health, sciatic nerve, peripheral nervous system diseases, magnetic resonance imaging, magnetic resonance neurography, machine learning, segmentation

## Abstract

Diagnosis of peripheral neuropathies relies on neurological examinations, electrodiagnostic studies, and since recently magnetic resonance neurography (MRN). The aim of this study was to develop and evaluate a fully-automatic segmentation method of peripheral nerves of the thigh. T2-weighted sequences without fat suppression acquired on a 3 T MR scanner were retrospectively analyzed in 10 healthy volunteers and 42 patients suffering from clinically and electrophysiologically diagnosed sciatic neuropathy. A fully-convolutional neural network was developed to segment the MRN images into peripheral nerve and background tissues. The performance of the method was compared to manual inter-rater segmentation variability. The proposed method yielded Dice coefficients of 0.859 ± 0.061 and 0.719 ± 0.128, Hausdorff distances of 13.9 ± 26.6 and 12.4 ± 12.1 mm, and volumetric similarities of 0.930 ± 0.054 and 0.897 ± 0.109, for the healthy volunteer and patient cohorts, respectively. The complete segmentation process requires less than one second, which is a significant decrease to manual segmentation with an average duration of 19 ± 8 min. Considering cross-sectional area or signal intensity of the segmented nerves, focal and extended lesions might be detected. Such analyses could be used as biomarker for lesion burden, or serve as volume of interest for further quantitative MRN techniques. We demonstrated that fully-automatic segmentation of healthy and neuropathic sciatic nerves can be performed from standard MRN images with good accuracy and in a clinically feasible time.

## 1. Introduction

Current state-of-the-art to diagnose and monitor the effects of potentially available treatments for peripheral neuropathies relies on clinical examination and electrodiagnostic studies (EDX). Certain regions of the body are less amenable to EDX, or may show ambiguous symptoms and signs regarding localization of lesions when affected: e.g., brachial and lumbosacral plexus, nerves situated deeply in the extremities such as the sciatic nerve, and nerves close to the trunk. Magnetic resonance neurography (MRN) (1, 2) has emerged as a complementary diagnostic instrument for peripheral neuropathy, especially where neurological examinations are difficult or inconclusive. While MRN has mostly been used as a qualitative diagnostic method, image-derived morphometric parameters (e.g., cross-sectional areas, CSA) and quantitative MR measures based on relaxometry, magnetization transfer, or diffusion-weighted imaging of peripheral nerves have increasingly been reported recently (3,–11), and could potentially serve as outcome measures (12).

Quantitative assessment of peripheral nerves from MRN typically proceeds either by assessment of CSA or by the identification of regions of interest in which abnormal signal behavior or quantitative MR parameters are further analyzed. In both cases, a segmentation of the peripheral nerve at interest is typically performed manually. It has been shown that for clinically relevant image-based quantification, fully- and semi-automatic computer-assisted segmentation is favorable to manual segmentation regarding reproducibility, time-efficiency, and cost-efficiency (13, 14). Computer-assisted segmentation of peripheral nerves from MRN images has been addressed by Felisaz et al. (15). They proposed a semi-automatic method to compartmentalize the tibial nerve in micro-neurography images and showed the potential of computer-assisted segmentation by associating peripheral neuropathy with decreased fascicular-to-nerve volume ratio, increased nerve volume, and increased CSA (16). Unfortunately, potential MR-based outcome measures of peripheral nerves will be of little clinical use if the assessment remains a manual, user-dependent, and tedious task for physicians or trained personnel, or is limited to a small field of view (FOV) as in Felisaz et al. (16). Fully- or semi-automatic computer-assisted segmentation of peripheral nerves with a large extremities coverage may be an important future step to obtain quantitative imaging outcome measures at a larger scale for disease-specific diagnosis and treatment monitoring of peripheral neuropathies.

We present, to the best of our knowledge, the first attempt of a fully-automatic, deep learning-based segmentation of peripheral nerves obtained from a larger coverage MRN image set of the thigh acquired in a clinical setting, containing healthy volunteers and patients suffering from peripheral nervous system (PNS) disorders.

## 2. Materials and methods

### 2.1. Healthy volunteer and patient data

All healthy volunteers and patients receiving a MRN examination at our institution between 2013 and 2017 have been enrolled in a registry. We developed and evaluated the proposed method on retrospectively chosen images of the human thigh from healthy volunteers, and all patients with clinically and electrophysiologically diagnosed sciatic neuropathy enrolled in our registry. The healthy cohort consisted of 10 volunteers (4 female, 6 male; age = 25.0 ± 2.6). The patient cohort consisted of 42 patients (21 female, 21 male; age = 55.7 ± 15.7), with sciatic neuropathy as confirmed by senior physicians (over 10 years of experience) in our neuromuscular disease unit based on clinical examination and EDX. The study was approved by the local ethical committee, and informed consent was obtained from all participants.

### 2.2. MR acquisition

For this retrospective study, a turbo spin-echo T2-weighted sequence without fat suppression (T2) was chosen, which is part of the routine MRN examination protocol at our institution. The sequence was acquired using either a circular 15-channel knee coil or an anterior body surface and posterior built-in spine coil in a clinical scanner running at 3 Tesla (Siemens MAGNETOM Verio, Siemens Healthcare GmbH, Erlangen, Germany) with following sequence parameters: repetition time (TR) of 4690 ms, echo time (TE) of 82 ms, FOV of 384 × 330 mm^2^, flip angle (FA) of 134 °, turbo factor of 12, voxel size of 0.52 × 0.52 × 4.0 mm^3^, and 60 axial-oriented slices with an inter-slice gap of 0.4 mm. The acquisition time was 4 min 43 s. Anatomical coverage was one image stack for each volunteer and patient, with image stacks positioned at the distal thigh in all volunteers, and variably between the distal thigh up to the head of the femur in patients, respectively.

### 2.3. Manual ground truth segmentation

Three physicians (experienced in clinical, electrophysiological, and imaging-based assessment of neuromuscular diseases: author OS, senior neurologist and neuroradiologist > 14 years, author BW, neuroradiologist > 4 years, and author LG, neuroradiologist > 2 years) manually segmented the sciatic nerve including its branches the tibial and peroneal nerve. Each physician individually segmented the available data to study the inter-rater agreement for manual sciatic nerve segmentation. A consensus segmentation (referred hereafter as consensus ground truth) for the evaluation of our computer-assisted segmentation approach was obtained using majority voting of the three rater segmentations, i.e., a voxel belongs to peripheral nerve if at least two raters segmented it. All segmentations were performed with the ITK-SNAP software^1^ (17) using the polygon or paintbrush tool.

### 2.4. Computer-assisted segmentation

**Figure 1:**
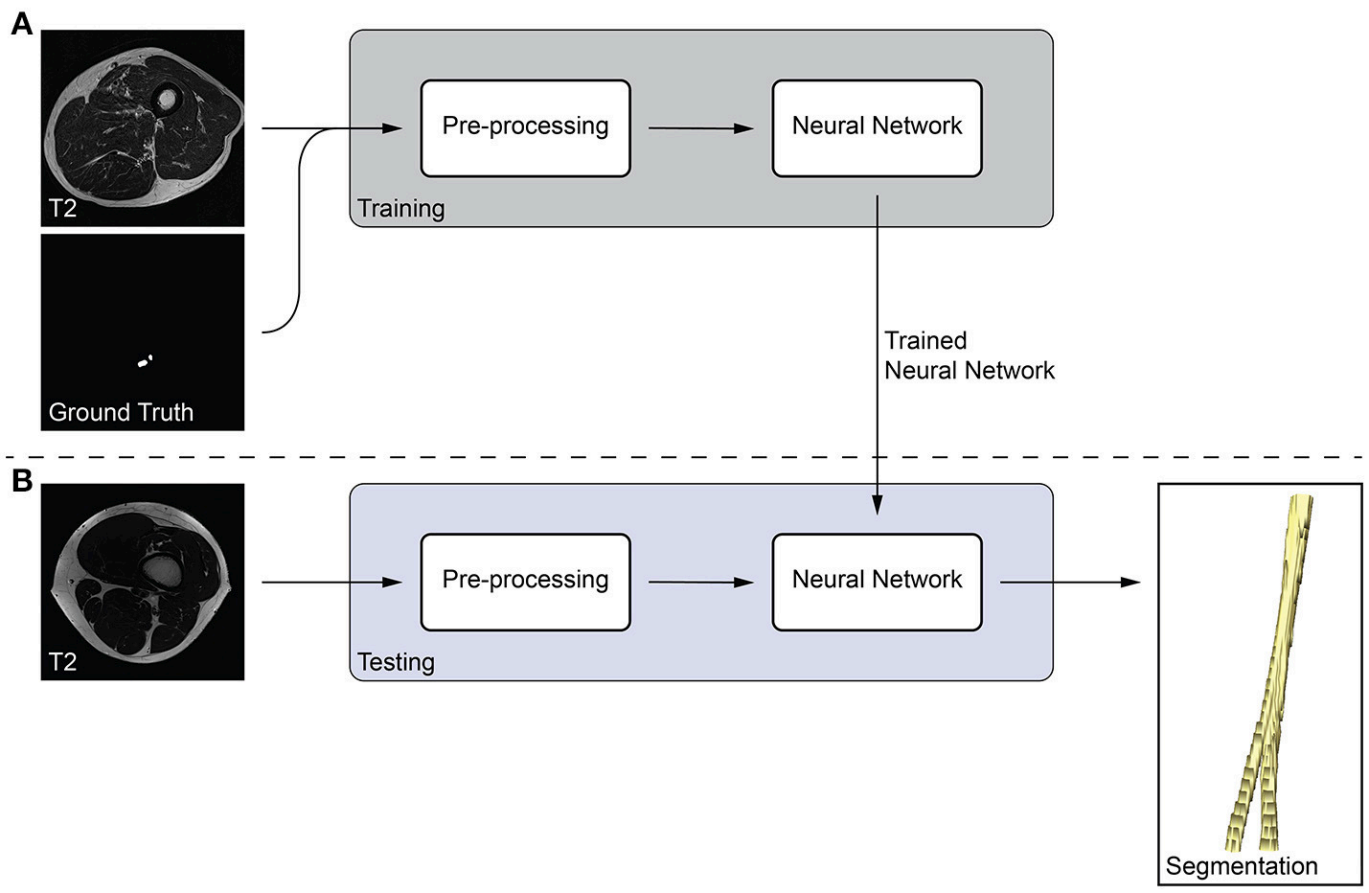
Overview of the proposed peripheral nerve segmentation method. **(A)** Training of the neural network with T2 and ground truth image slices, and **(B)** testing of the trained neural network yields a segmentation of the peripheral nerve without the need of a ground truth.

We developed a fully-automatic, deep learning-based approach to segment peripheral nerves from MRN images. The input of our method was the T2 image, and the output of the method was a binary segmentation of the T2 image into peripheral nerve and background. Our method bases on fully-convolutional neural networks (fCNNs), often referred to as deep learning algorithms or simply as neural network, which have shown excellent performances in various medical image segmentation tasks (18). The main working principle behind fCNNs is supervised learning, a machine learning paradigm in which a computer learns to distinguish peripheral nerve from background tissue using training data (Figure 1A). In our case, the training data consisted of T2 images as well as the corresponding consensus ground truth images. Our fCNN processed the images slice-wise, i.e., the input was a T2 image slice, and the output was a segmented binary mask of the T2 image slice. This segmented image slice was then compared to the image slice of the consensus ground truth to improve the segmentation during the training of the fCNN. Once a fCNN is trained, new and previously unseen images can be segmented without the need of any ground truth image (Figure 1B). During this so-called testing of the fCNN, the neural network segments a T2 image slice into peripheral nerve and background without the need for a manually segmented ground truth image slice. Segmenting all T2 image slices resulted in an entirely segmented peripheral nerve in the T2 image. The following sections describe the pre-processing of our data, the neural network, and our training strategy.

#### 2.4.1. Pre-processing

We pre-processed the T2 images by intensity normalization, and cropping. First, we normalized the intensities of each T2 image to zero mean and unit variance. Second, we cropped each T2 image to 320 × 320 pixels in the axial plane to be able to reduce the image size evenly, which is a requirement of our neural network architecture.

#### 2.4.2. Neural network

Our fCNN architecture adopts the fully-convolutional DenseNet (19). It consists of four transition down (TD) in the downsampling path and four transition up (TU) in the upsampling path. We defined a layer in our neural network to be the following sequence of operations: batch normalization (20), rectified linear unit (ReLU) activation function, 3 × 3 convolution with stride one and same padding, and dropout (21) with probability *p* = 0.2. Our dense blocks consist of four consecutive layers each with 12 filters (growth rate 12). A TD applied batch normalization, ReLU, 1 × 1 convolution with stride one and same padding, dropout with *p* = 0.2, and 2 × 2 max pooling with stride two. Therefore, the input resolution was 20 × 20 pixels in the bottleneck dense block. A TU applied a transposed 3 × 3 convolution with stride two and same padding. Before the first dense block in the downsampling path, a 3 × 3 convolution with stride one, same padding, and 48 filters was applied to the input. Similarly, we applied a 1 × 1 convolution with stride one, same padding, and two filters, the desired number of classes, after the last dense block in the upsampling path. Finally, a softmax non-linearity was applied to calculate the pixel-wise probability distribution for background and peripheral nerve. The fCNN was implemented using PyTorch 0.4.0 (Facebook, Inc., Menlo Park, CA, U.S.) with Python 3.6 (Python Software Foundation, Wilmington, DA, U.S.).

#### 2.4.3. Training

We trained the neural network on the 10 healthy volunteers and 42 patient images using a randomly generated four-fold cross-validation. That is, the network was trained on 39 images, and its performance was tested on 13 images. This procedure was repeated for all four folds, such that the network's performance on each image could be assessed. During the fold randomization, we balanced healthy volunteer and patient images (i.e., we ensured that each fold contained at least two volunteer images among the 13 test images). For training, we used a cross entropy loss and the Adam optimizer (22) with a learning rate of 1 × 10-3, β_1_ = 0.9, and β_2_ = 0.999. We decreased the learning rate to 1 × 10-4 after 30 epochs, and to 1 × 10-5 after 80 epochs. The training was run with a batch size of eight image slices for 100 epochs, which we empirically found to be sufficient. Additionally, we used data augmentation during the training to prevent memorization of training data and to introduce artificial variety: random flipping, random translation, and random elastic deformation. Note that we tuned the neural network on one randomly chosen cross-validation split and did not use the other splits to develop and tune the method.

### 2.5. Evaluation and statistical analysis

We compared the performance of our method to the consensus ground truth (Auto-GT), and additionally to the inter-rater variability (R-R). The inter-rater variability quantifies the difficulty of peripheral nerve segmentation and served as baseline for our method. It was obtained by comparing the manual segmentations of each possible rater-rater pair (i.e., OS-BW, OS-LG, and BW-LG) and aggregating these comparisons per cohort [cf. Section III-E in (23)]. Therefore, the inter-rater variability consisted of *n* = 30 (healthy volunteer cohort) and *n* = 126 (patient cohort) results for every evaluation metric.

We used the following three evaluation metrics to evaluate the performances: (1) The Dice coefficient (24), which measures the spatial overlap between the segmentation and the ground truth. (2) The Hausdorff distance (HD) (25), which measures the 95th percentile distance between the segmentation and the ground truth boundaries. (3) The volumetric similarity (VS) (26), which measures the absolute difference between the segmentation and ground truth volume divided by the sum of the two volumes. We used the open-source evaluation metrics implementation presented in Taha and Hanbury (27) (version 2017.04.25) and refer the reader to the reference for mathematical details. Furthermore, we measured the execution time of our method and the raters' segmentation time .

We hypothesized that there is no statistically significant difference between our method (Auto-GT) and the inter-rater baseline (R-R). The hypothesis was tested for both cohorts and the three evaluation metrics independently. We used an unpaired Mann-Whitney *U*-test to confirm the hypothesis due to non-normal distributed data and different sample size between Auto-GT and the aggregated R-R results. All tests were performed with a significance level of 0.05 (95 % confidence interval) using R (R Core Team, Vienna, Austria) version 3.5.0.

## 3. Results

**Figure 2:**
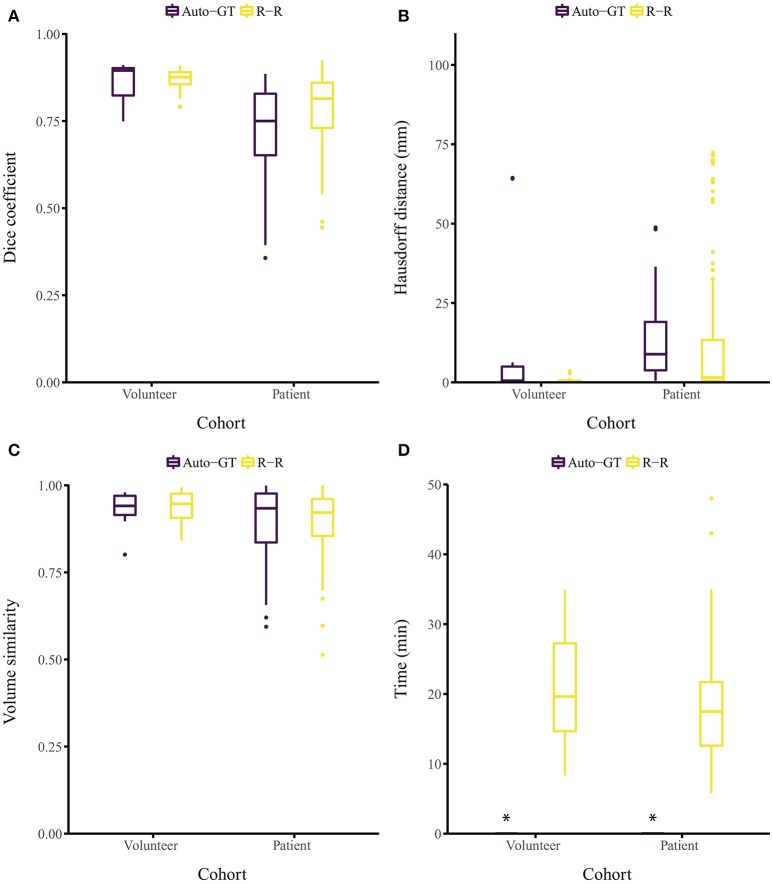
Segmentation evaluation metrics of our method (Auto-GT) compared to inter-rater variability (R-R). Boxplot of the **(A)** Dice coefficient, **(B)** Hausdorff distance, **(C)** volume similarity, and **(D)** segmentation time separated by healthy volunteer and patient cohort. *The segmentation time of our method is less than 1 s and therefore barely visible in the boxplot.

The proposed approach yielded Dice coefficients of 0.859 ± 0.061 and 0.719 ± 0.128 for the healthy and patient cohorts, respectively. Regarding the HD metric, the approach yielded 13.9 ± 26.6 and 12.4 ± 12.1 mm, respectively. Finally, the VS metric resulted in 0.930 ± 0.054 and 0.897 ± 0.109, respectively. The inter-rater performance was 0.869 ± 0.031 and 0.786 ± 0.093 for the Dice coefficient, 0.70 ± 0.67 and 11.2 ± 19.0 mm for the HD, and 0.937 ± 0.043 and 0.897 ± 0.087 for the VS. Overall, for each metric, the segmentation performance was better for the healthy volunteer cohort than the patient cohort (Figures 2A–C). No statistical significant differences (*p* > 0.05) between Auto-GT and R-R were found for the volunteer Dice coefficients (*p* = 0.6), the volunteer VS (*p* = 0.8), and the patient VS (*p* = 0.3). Statistical significant differences (*p* < 0.05) were found for patient Dice coefficients (*p* = 0.002), volunteer HD (*p* = 0.02), and patient HD (*p* ≤ 0.001). The detailed results of the segmentation analysis between individual raters, the consensus ground truth, and our proposed approach for both cohorts are summarized in Table 1.

**Table 1:** Detailed segmentation evaluation metrics for both healthy volunteer and patient cohorts.

**Cohort**	**Comparison**	**Dice coefficient**	**Hausdorff distance (mm)**	**Volume similarity**
Volunteer	OS-BW (*n* = 10)	0.865 ± 0.018	0.521 ± 0.000	0.917 ± 0.038
	OS-LG (*n* = 10)	0.878 ± 0.034	0.749 ± 0.722	0.973 ± 0.024
	OS-GT (*n* = 10)	0.938 ± 0.015	0.469 ± 0.165	0.964 ± 0.015
	BW-LG (*n* = 10)	0.862 ± 0.038	0.840 ± 0.935	0.922 ± 0.044
	BW-GT (*n* = 10)	0.927 ± 0.021	0.417 ± 0.220	0.951 ± 0.030
	LG-GT (*n* = 10)	0.936 ± 0.034	0.541 ± 0.836	0.970 ± 0.025
	R-R (*n* = 30)	0.869 ± 0.031	0.703 ± 0.672	0.937 ± 0.043
	Auto-OS (*n* = 10)	0.850 ± 0.057	13.9 ± 26.5	0.950 ± 0.057
	Auto-BW (*n* = 10)	0.830 ± 0.078	12.2 ± 23.3	0.897 ± 0.075
	Auto-LG (*n* = 10)	0.834 ± 0.052	14.1 ± 26.3	0.934 ± 0.052
	Auto-GT (*n* = 10)	0.859 ± 0.061	13.9 ± 26.6	0.930 ± 0.054
Patient	OS-BW (*n* = 42)	0.807 ± 0.088	8.68 ± 15.7	0.890 ± 0.073
	OS-LG (*n* = 42)	0.784 ± 0.074	10.2 ± 16.2	0.909 ± 0.076
	OS-GT (*n* = 42)	0.906 ± 0.040	2.02 ± 4.56	0.939 ± 0.041
	BW-LG (*n* = 42)	0.766 ± 0.112	14.9 ± 23.9	0.893 ± 0.107
	BW-GT (*n* = 42)	0.896 ± 0.073	4.99 ± 13.4	0.942 ± 0.069
	LG-GT (*n* = 42)	0.870 ± 0.070	7.11 ± 15.5	0.939 ± 0.064
	R-R (*n* = 126)	0.786 ± 0.093	11.2 ± 19.0	0.897 ± 0.087
	Auto-OS (*n* = 42)	0.695 ± 0.137	13.9 ± 13.4	0.868 ± 0.121
	Auto-BW (*n* = 42)	0.690 ± 0.139	15.9 ± 14.6	0.878 ± 0.117
	Auto-LG (*n* = 42)	0.678 ± 0.126	14.7 ± 13.3	0.886 ± 0.119
	Auto-GT (*n* = 42)	0.719 ± 0.128	12.4 ± 12.1	0.897 ± 0.109

*The comparisons are: rater pairs (OS-BW, OS-LG, BW-LG), rater to consensus ground truth (OS-GT, BW-GT, LG-GT), inter-rater variability (R-R), method to raters (Auto-OS, Auto-BW, Auto-LG), and method to consensus ground truth (Auto-GT). Values are given as mean ± standard deviation*.

A *post-hoc* analysis revealed that the distribution of image stack location along the thigh was more variable in patients than in healthy volunteers. For healthy volunteers, slice stacks were predominantly taken from the mid-thigh, while in patients the stacks were variously taken from the distal, medial, and proximal portions of the thigh. To analyze the location dependence of the segmentation, we subdivided the patient cohort into locations distal, medial, and proximal thigh, with medial being approximately at the same location as the healthy volunteer cohort. Dice coefficients were 0.687 ± 0.144 for distal thigh (*n* = 14), 0.720 ± 0.109 for medial thigh (*n* = 7), and 0.741 ± 0.123 for proximal thigh (*n* = 21) in the patient cohort.

**Figure 3:**
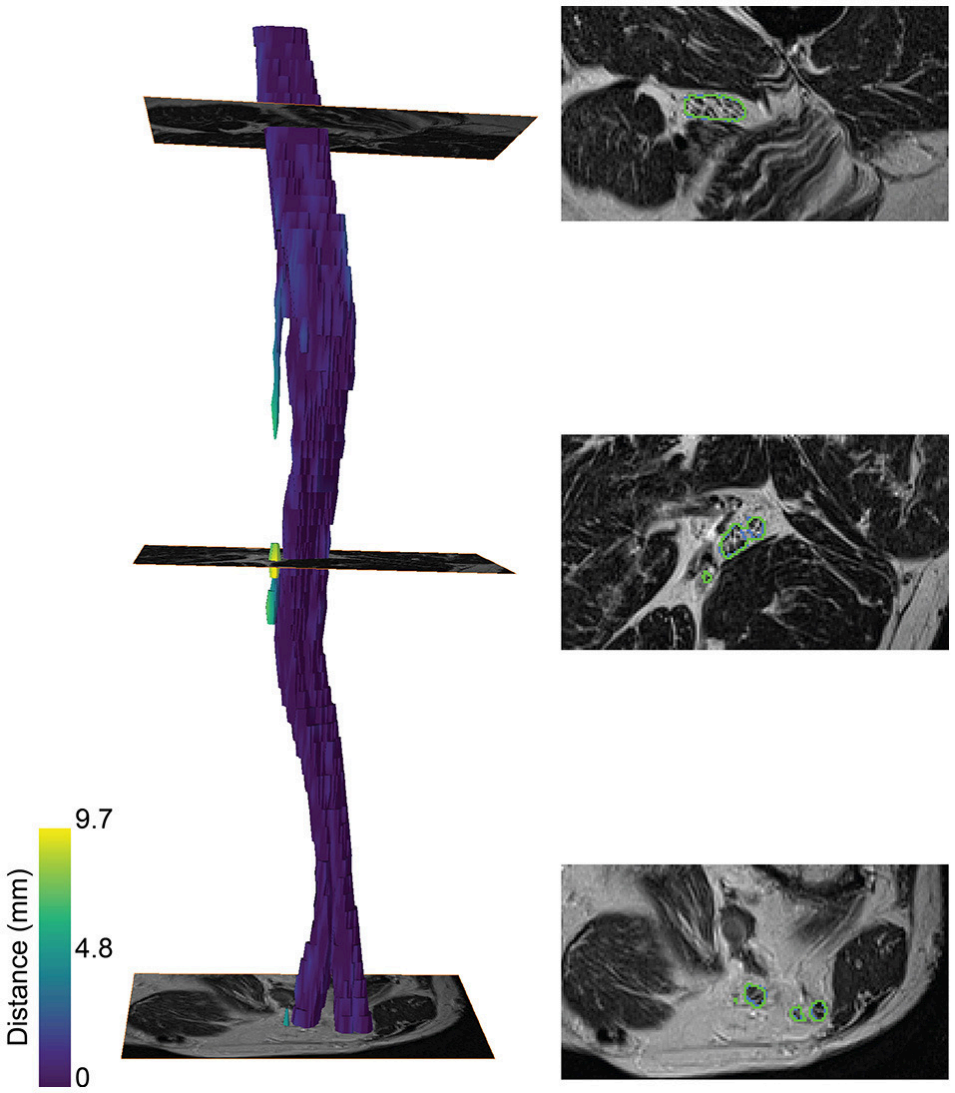
Segmentation of the sciatic nerve of a patient. **(Left)** 3-dimensional rendering of the segmentation. The color map encodes the surface-to-surface distance of the segmentation to the ground truth. **(Right)** The segmentation boundaries (green) and ground truth boundaries (blue) on the T2 image are shown for three slices along the nerve course.

A color-coded three-dimensional rendering of a segmented sciatic nerve for a representative patient result, showing the similarity between the segmentation and the ground truth, is depicted in Figure 3. A higher surface-to-surface distance is present at over-segmented locations as can be seen on the T2 image sections with the segmentation (green) and ground truth boundaries (blue).

Segmenting the image stack of one subject with our method on a standard desktop computer equipped with a graphics processing unit (GPU) required less than 1 s for the volunteer and the patient cohort (Ubuntu 16.04 LTS, 3.2 GHz Intel Core i7-3930K, 64 GB memory, NVIDIA TITAN Xp with 12 GB memory). The time required to segment the ground truth of one subject manually was 21.1 ± 7.68 and 18.2 ± 7.42 min for the volunteer and patient cohort, respectively (Figure 2D). Note that the one-time training (without any user interaction) of the fCNN before segmenting new subjects required approximately 5 h.

## 4. Discussion

A fully-automatic, deep learning-based segmentation of peripheral nerves for T2-weighted MRN images was evaluated on thigh scans acquired in a clinical setting. The proposed method was successful in segmenting peripheral nerves with and without lesions with good accuracy both in healthy volunteers and patients suffering from sciatic neuropathy. Our fully-automatic method results in a significant time gain for sciatic nerve segmentation, compared to manual segmentation.

**Figure 4:**
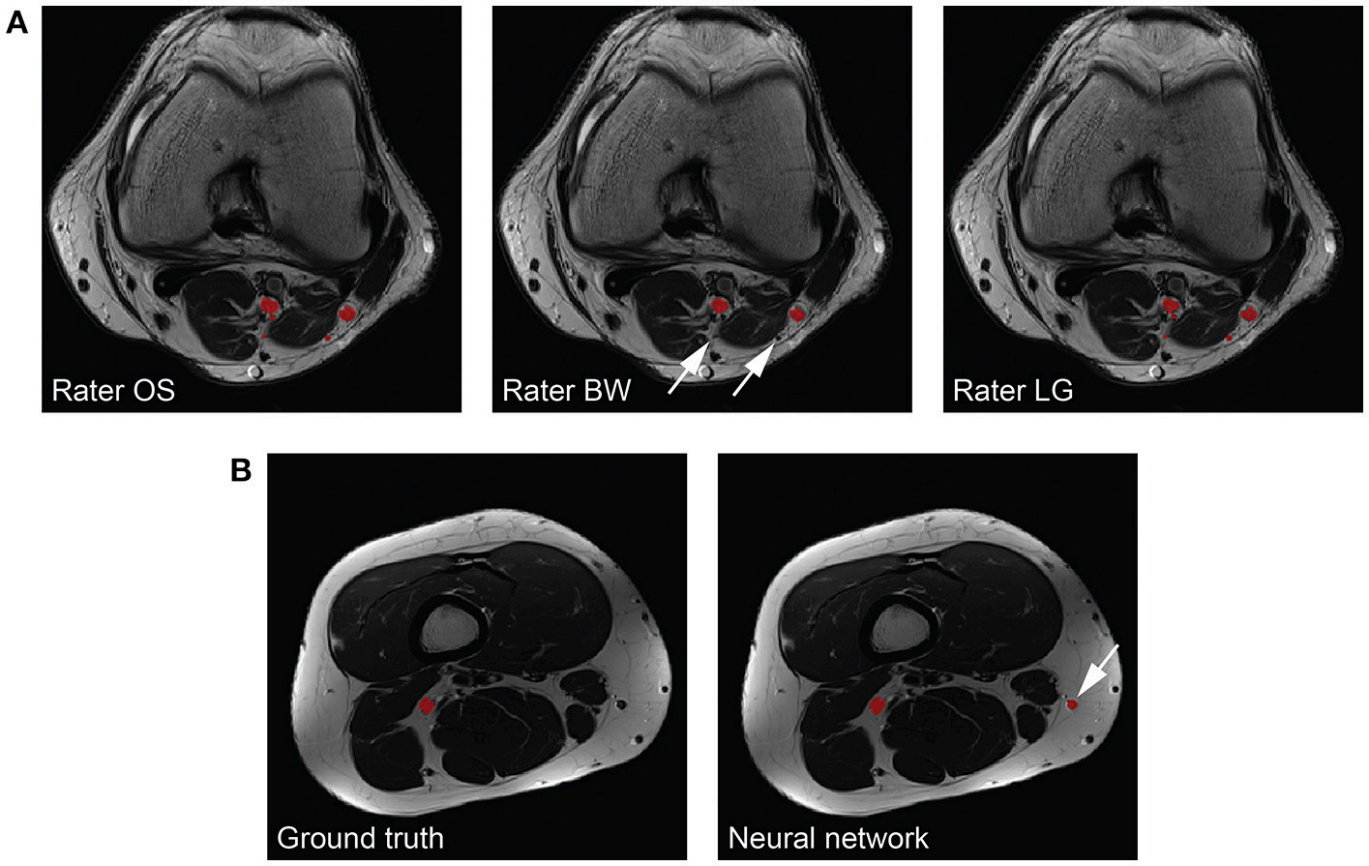
Interpretability of the Hausdorff distance (HD) metric for peripheral nerves. **(A)** The same nerves segmented by the three raters (left to right) are depicted in red. One rater does not segment all branches (arrows), which results in a large HD. **(B)** The consensus ground truth (left) compared to the segmentation results by our method (right). A falsely segmented vein (arrow) by our method results in a large HD.

The peripheral nerves in our images can be considered as small structures with a volume fraction (i.e., nerve volume divided by background volume) of 0.143 ± 0.049 %. Unfortunately, evaluating the segmentation performance of methods applied to small structures is difficult because commonly used metrics such as the Dice coefficient and the VS are sensitive to small structures (27). While distance metrics are more suitable for evaluating small structures, they might not always be of interest to clinicians as they can be sensitive to outliers (27). Our inter-rater study revealed that our method reaches human level performance for volunteer Dice coefficients, and VS in both healthy volunteers and patients. Inter-rater disagreement for small cutaneous branches of the tibial and peroneal nerve was present in patients mostly because of more distally located image stacks in this cohort, whereas in our healthy volunteers the cutaneous branches were mostly not branching from the tibial or peroneal nerves yet. Missing such a branch on one or several image slices results in a higher mean and standard deviation of the HD metric compared to the volunteer cohort (see Figure 4A for an example). Furthermore, large HD metrics arise in the case of false positive segmentations of our method. A volunteer example with a Dice coefficient of 0.772 but a HD of 64.1 mm is shown in Figure 4B, where a falsely segmented vein contributes to the large HD. Overall, interpreting the segmentation performance for peripheral nerves is challenging, especially in the case of the HD metric, and may be misleading when comparing metrics to other segmentation tasks such as brain tumors.

Our approach has several limitations and difficulties, which resulted in a slightly decreased segmentation accuracy for the patient cohort. The difference in performance between the two cohorts is mainly assumed to be caused by the variability in PNS appearance, differences in the image quality and location of the image stacks. While the volunteers were imaged with a standardized positioning protocol for MRN sequence quality assurance, the patient images were obtained retrospectively from a clinical setting for which no standardized protocol was used. Consequently, different peripheral nerve lesion types and muscle pathologies were present in the images, and the position of the MR acquisition differed considerably, which also might cause the image stack to no longer be perpendicular to the main nerve direction (e.g., in proximal parts of the thigh near the hip or at the knee region). Further, the contrast between peripheral nerve and its surrounding tissues was sometimes very limited on distal image slices. These aspects ultimately increase the complexity of the segmentation task, which is reflected in the lower Dice coefficients found for distally located regions on patient images, as compared to the regions located proximally or at mid-thigh in volunteer and patient images. Besides increasing the amount of training data, a potential workaround could be the use of isotropic 3-D T2-weighted sequences for MRN despite their lower resolution [e.g., SPACE, Sampling Perfection with Application optimized Contrasts using different flip angle Evolution (28)]. In addition, the quality of the patient images was lower than the quality of the volunteer images. Movement artifacts of varying strength were noticeable mostly in the patient cohort due to uncomfortable scanning positioning, while the volunteers could be imaged without noticeable movement artifacts. More frequently present in the patient cohort were also signal distortion artifacts, stemming from an off-center positioning of the extremities during the MR acquisition. Due to the retrospective type of study performed, healthy volunteers and patient cohorts differed regarding mean age, and detailed clinical data on the patient cohort was not available. Hence, we can neither rule out an effect of age, nor of severity of the neuropathy on segmentation performance.

**Figure 5:**
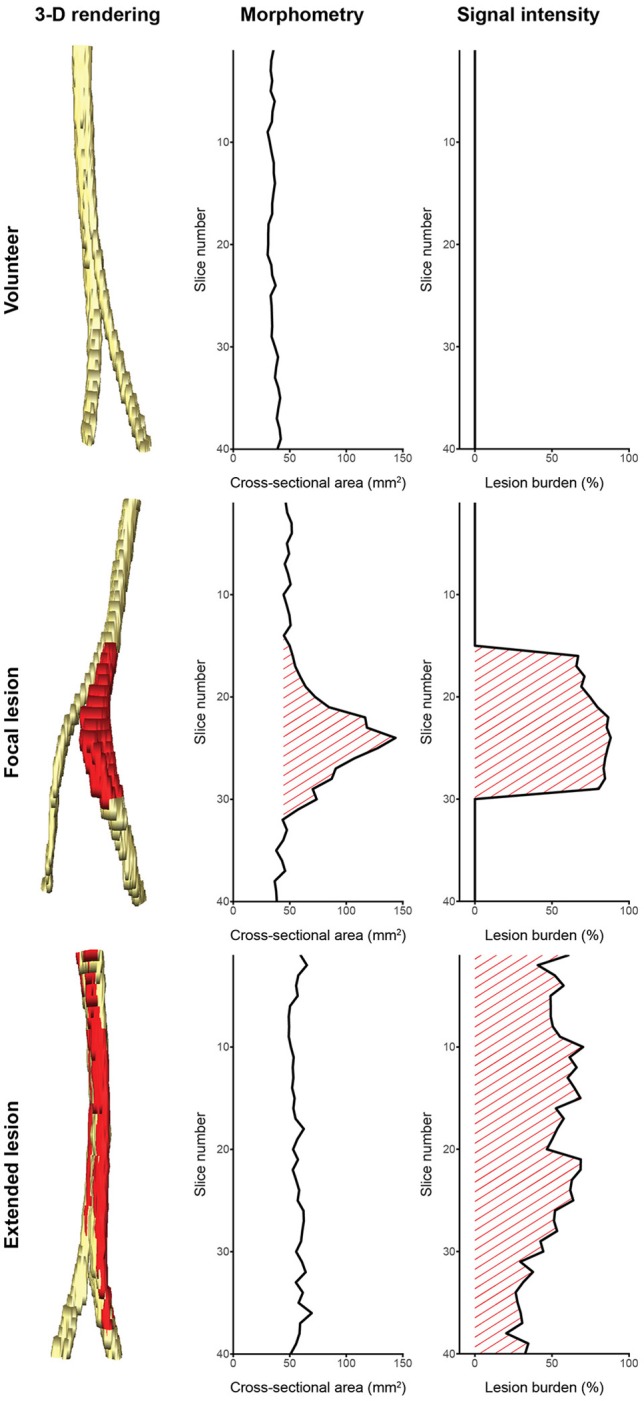
Potential of computer-assisted segmentation of peripheral nerves for imaging biomarkers. (**Left** column) 3-dimensional renderings of the sciatic nerve with lesions colored red: (**Top** row) a healthy volunteer, (**Middle** row) a patient with a focal lesion, (**Bottom** row) and a patient with an extended lesion. (**Middle** column) Cross-sectional area evolution and (**Right** column) lesion burden evolution obtained from the segmentation could be used as biomarkers to assess disease severity and progression, or to categorize the lesion type. Note that not all peripheral nerve lesion types show morphometric abnormalities, hence a combination with signal intensity (or other quantifiable MR parameters) is necessary to assess the lesion burden. The quantified signal intensity evolution was assessed by segmenting hyperintense nerve fascicle bundles on a co-registered T2-weighted sequence with fat suppression using inversion recovery.

We see our contribution as a proof of concept for segmentation of peripheral nerves for a wider clinical usage of quantitative MRN as an adjunct to clinical assessment and EDX, especially in more proximal body regions not well accessible using aforementioned standard work-up. Despite the discussed limitations, the partially achieved human-level performance in this retrospective analysis of MRN images demonstrates the promising usefulness and strength of fully-automatic segmentation. This is additionally emphasized by the fact that achieving a high agreement of a manual segmentation to a consensus ground truth requires experienced personnel with a long training history (see better results of rater OS in Table 1), while partially achieved human-level performance of the fCNN required just 5 h of unassisted training. We think that such peripheral nerve segmentation is essential toward obtaining quantitative imaging outcome measures, which could be an integral part of MRN-based examinations of PNS and PNS disorders, both for diagnostic and monitoring purposes. Our method could assist clinicians by providing a quantification of nerve volume, CSA, and lesion burden through a combination of morphometry and signal intensity analysis as shown in Figure 5. Such analyses might allow distinguishing between healthy nerve and diseased nerve, and peripheral neuropathy type as it has been shown by others (5, 7–11). Additionally, our segmentation could serve as a volume of interest for other quantitative MR methods like diffusion-weighted imaging, magnetization transfer imaging, and relaxometry (3–8, 10), which might further be applied to assess total lesion burden, distribution pattern of lesions, and multi-model characterization of lesions. However, such potential outcome measures will only find acceptance in a clinical setting, if they can be obtained in an accurate and time-efficient manner without tedious and labor-intensive manual segmentation.

Further refinements of the method could include algorithmic as well as MRN related changes. Algorithmic changes could include three-dimensional convolutions to enrich the contextual information during the segmentation. Other than that, a dedicated post-processing of the segmentation could reduce false positive segmentation by reconstructing the peripheral nerves under physiological constraints similar to Rempfler et al. (29). Regarding changes in MRN, additional MRN images could be used to enrich the information about the peripheral nerves, especially for regions showing problems with fully-automated segmentation. Also, combining the complementary information from MRN images with diffusion-weighted images (e.g., from tractography of peripheral nerves) may add additional imaging information for better discrimination of peripheral nerves from surrounding tissue [e.g., (4, 30)]. In general, the segmentation could be extended to peripheral nerves of other body regions, to peripheral nerve lesions, and to muscles (31) with the aim of a holistic computer-assisted quantification of neuromuscular diseases.

In conclusion, we proposed, to the best of our knowledge, the first fully-automatic, deep learning-based segmentation of peripheral nerves from the thigh in a clinical setting. Our method segments healthy and diseased peripheral nerves from MRN images with good accuracy and in clinically feasible time, and is a promising approach toward quantitative outcome measures for the diagnosis of peripheral neuropathies.

## Ethics statement

This study was carried out in accordance with the recommendations of the Swiss legislation for human research. All subjects gave written informed consent. The protocol was approved by the local ethics committee of the Canton of Bern, Switzerland.

## Author contributions

FB, WV, MR, and OS conceived the ideas and designed the study. FB developed the method and analyzed the results. CS, MA, BW, and OS contributed to data collection and registry enrollment. BW, LG, and OS manually segmented the ground truth. ME-K supervised the work. FB, WV, MR, and OS contributed to manuscript writing. All authors revised the manuscript. OS is the scientific guarantor of the study.

### Conflict of interest statement

The authors declare that the research was conducted in the absence of any commercial or financial relationships that could be construed as a potential conflict of interest.

## Footnotes

^1^Available from www.itksnap.org
